# Burnout and its associated factors among medical students of Jazan University, Jazan, Saudi Arabia

**DOI:** 10.1108/MIJ-06-2020-0011

**Published:** 2020-11-30

**Authors:** Mohamed Saih Mahfouz, Suhaila Abdalkarim Ali, Haya Ahmed Alqahtani, Amani Ahmad Kubaisi, Najla Mohammed Ashiri, Eshrag Hassan Daghriri, Shaima Ali Alzahrani, Azhar Ahmed Sowaidi, Afnan Mousa Maashi, Doa’a Albarag Alhazmi

**Affiliations:** Department of Family and Community Medicine, Faculty of Medicine, Jazan University, Jazan, Saudi Arabia; Faculty of Medicine, Jazan University, Jazan, Saudi Arabia

**Keywords:** Burnout, Copenhagen burnout inventory, Jazan

## Abstract

**Purpose:**

The purpose of this study is to assess the prevalence of burnout syndrome and its associated factors among medical students at Jazan University, Jazan, Kingdom of Saudi Arabia.

**Design/methodology/approach:**

A cross-sectional survey was conducted among 440 randomly selected medical students at Jazan University. The questionnaire used for this study was based on the Copenhagen Burnout Inventory.

**Findings:**

The overall prevalence of burnout was estimated at 60.2% (95% CI 55.6–64.8). The prevalence was higher for females (64.1%) than for males (56.2%) but without statistically significant differences (*p* > 0.05). On average, the students scored the highest averages in the personal burnout category, followed by the study-related and client-related burnout categories. In the multivariate analysis, a lower age (beta = −3.17, *p* = 0.026), female (beta = −0.896, *p* = 0.016), and having better burnout knowledge (beta = 0.710, *p* = 0.025) predict significantly higher personal burnout.

**Practical implications:**

It is necessary to implement strategies to reduce the incidence of burnout among medical students for the sake of a better quality of life for future doctors.

**Originality/value:**

There is a high prevalence of burnout among Jazan’s medical students.

## Background

Medical education at the undergraduate level is associated with increased stress and depression among the students (Alharbi *et al.*, 2018; Sarkar *et al.*, 2017; Sani *et al.*, 2012; Abdulghani, 2008; El-Gilany *et al.*, 2008). The long process of the study modules and the academic environment have multiple challenges to students and expose them to increasing stress.

The sources of stress in medical training are diverse, ranging from keeping appropriate academic achievements to the vastness of the educational curriculum (Sani *et al.*, 2012; Abdulghani, 2008; El-Gilany *et al.*, 2008). The continuous exposure to stress without taking a break may lead to a decrease in achievements ability, and this, in turn, causes the burnout syndrome (Moghadam *et al.*, 2017).

Burnout syndrome is a state of emotional, mental and physical exhaustion caused by excessive and prolonged stress (Schaufeli *et al.*, 2002). It occurs when the person feels overwhelmed, emotionally drained and unable to meet constant demands (Moghadam *et al.*, 2017; Schaufeli *et al.*, 2002).

Many studies have been conducted internationally (Moghadam *et al.*, 2017; Schaufeli *et al.*, 2002; Asencio-López *et al.*, 2016; Muzafar, *et al.*, 2015; Costa *et al.*, 2012) and at KSA level (Albalawi *et al.*, 2015; Almalki *et al.*, 2017; Chang *et al.*, 2012; Aboalshamat *et al*, 2017; Altannir *et al.*, 2019) showed that medical students are borne to various degrees of burnout. Burnout among students has three dimensions: first emotional, fatigue cynicism and low professional efficacy (Carlotto and Câmara, 2006). Increasing shreds of evidence suggested the effect of burnout syndrome on the academic performance of medical students (Rana, 2016). Further, it illustrated that burnout is associated with psychiatric disorders and suicidal ideation (Dyrbye, *et al.*, 2008).

Although there is increasing global interest (IsHak *et al.*, 2013) in the burnout syndrome, no previous study assessed the extent of burnout among medical students at Jazan University.The main objective of this study was to evaluate the prevalence of burnout syndrome and its associated factors among medical students in Jazan University.

## Research methods

### Study design, area and population

This research used an analytical cross-sectional study design. The research was conducted in the Faculty of Medicine at Jazan University. Jazan is located in the southwest of the Kingdom of Saudi Arabia (KSA) on the Red Sea. Study participants were medical students at Jazan University, who registered for the academic year (2017–2018).

### Sampling procedures

The sample size is calculated to be 440 students. The estimation was based estimated on sample size formula for cross-sectional study design, using the following parameters response *p* = 50% (As no previous study on Burnout conducted at Jazan University), 95% confidence interval and error, not more than 5%. Also, the study assumed a non-response rate of 10%:

$$n=\frac{Z^{2}\;P(1-P)}{d^{2}}\;$$

The sampling design will be divided into males and females and stratified according to different classes within the faculty of Medicine at Jazan University all.

## Data collection and study tool

Data was collected by using a self-administered questionnaire. The study mainly used the Copenhagen Burnout Inventory (CBI) questionnaire (Kristensen *et al.*, 2005), which measures the three aspects of burnout, namely, personal, work related and patient related and consists of 19 questions in the 3 mentioned domains.

Personal domain refers to the state of prolonged physical and psychological exhaustion. Work-related burnout relates to the degree of physical and mental fatigue and exhaustion that is perceived by the person as related to his or her study. We defined a client-related burnout, as the degree of physical and psychological fatigue and exhaustion that is perceived by the student as related to his or her work with clients. The clients are a broad concept covering terms such as colleagues, patients, inmates, students, residents, etc.

The validity, reliability of the CBI has been previously discussed, and the Cronbach’s alpha for the burnout scale was estimated at 0.87 (Kristensen *et al.*, 2005; Milfont *et al.*, 2008). Students responses were made in the following categories: always, often, sometimes, seldom and never; the corresponding scores for each category are 100, 75, 50, 25 and 0, respectively. For each aspect of the burnout (personal, work related and patient related), average score was calculated. Free or minimal is defined as average total scores less than or equal 50, high burnout average more than 50. In addition to the CBI questionnaire, the study collected background information on the study participants including, gender, age, cumulative grade points average (CGPA), marital status and mode of living.

## Data management and analysis

Data collected was checked on regularly bases by the study team and entered into SPSS program for analysis. Descriptive statistics was used first to summarize the data using frequency distributions, graphs, means, etc. To compare categorical variables, chi-square test was used. Linear regression models were used to assess the relationship between the depended variable – burnout as a continuous variable with potential predictors such as age, gender, burnout level of knowledge, level of studies, CGPA and some selected preventive measures. A *p*-value < 0.05 was used to indicate statistical significance.

## Ethical consideration

This study was conducted in accordance with ethical standards of the KSA. All the participants read, understood and signed a written consent form.

The anonymity of participants was emphasized, and confidentiality was strictly maintained on all collected questionnaires. Finally, the study was approved by the ethical Committee of Jazan University (HAPO-10-Z-001) (Approval #REC39/8-S024).

## Results

Of the 440 college students recruited, 438 completed the questionnaires, with a response rate of 99.5.0%. A total of 233 (53.2%) were females, with a mean age of 21.91. ±1.6 years. Sociodemographic characteristics of the study population are described in Table 1. Regarding the marital status of the students, a majority of them were singles and rural students represented (53.2%) of the total number of the students.

Students’ knowledge regarding burnout showed that 26.7% had low knowledge scores, and that 40.6% had intermediate knowledge, whereas 32.7% reported a high level of knowledge scores (Table 2). The difference in the knowledge scores by age group showed no statistical significance (*p* = 0.166). Students from urban areas had a better knowledge score than those from rural areas, but the difference was not statistically significant (*p* = 0.512). Females had reported higher knowledge scores (38.3%) than males (26.6%); this difference was statistically significant (*p* < 0.05) (Table 2).

Burnout scores were categorized to free/minimal and significant (high) burnout. Based on this categorization, the overall prevalence of burnout was estimated at 60.2% (95% CI 55.6–64.8) higher for females (64.1%) than for males (56.2%) but without significant difference (*p*> 0.05). Regarding academic year, the high level of burnout among the students was reported among students of the fifth year (68.2%) and the lowest in the sixth year (internship 54.2%) also without statistical significance (*p*> 0.05). Students from Urban areas were characterized by having a high level of burnout (64.1%) compared with those from rural areas (58.1%) with statistical no significance difference (*p* > 0.05) (Table 3).

Table 4 presents descriptive statistics (mean and SD) on each of the burnout dimensions, according to some selected characteristics. On average, students scored the highest averages in the personal burnout category, followed by study-related burnout and client-related type yielding lower scores, for client-related burnout males scored a higher mean (44.7) than females (37.6) with statistically significant difference (*p* = 0.002).

Table 5 illustrates the students’ responses on preventive measures regarding the burnout. More than 50% of the respondents had never or rarely enjoyed a good vacation; however, 24.9% reported that they have a good vacation. About one-quarter of the respondents never practice any exercise for at least one hour, whereas 1.8% said that they never did anything for fun. About 15% of the studied sample had never felt a healthy life, whereas only 10.4% always having a full day to do their lovely things. A total of 20% of the studied sample rarely have a good sleep quality for 8–9 h per night.

In multivariate analysis (Table 6) decreasing age (Beta = −3.17, *p* = 0.026), being a female (Beta = −0.896, *p* = .016); and increase burnout knowledge (Beta = 0.710, *p* = 0.025) predict significantly higher personal burnout. Also, increase in the burnout knowledge (Beta = 0.277, *p* =0 .000), academic year (Beta = 0.175, *p* = 0.000) and CGPA (Beta = 0.369, *p* = 0.000) significantly predict high study-related burnout. Age (0.762 = 0.369, *p* = 0.002) and CGPA (Beta = 0.305, *p* = .037) significantly predict high client-related burnout.

## Discussion

To the best of our knowledge, this is the first study to deal with burnout syndrome at Jazan University. This study attempts to assess the prevalence of burnout syndrome and its associated factors among medical students. The results revealed a worrisome prevalence of burnout of 60.2% among all medical students from second to sixth year.

Consulting the literature on medical students’ burnout (Albalawi *et al.*, 2015; Almalki *et al.*, 2017; Aboalshamat *et al.*, 2017; Altannir *et al.*, 2019; Al-Alawi *et al.*, 2019; Stein and Sibanda, 2016; Chin *et al.*, 2016; Atlam, 2018; Fares *et al.*, 2016a; Popa-Velea *et al.*, 2017 and DeWitt *et al.*, 2016). indicate the use of different scales to measure burnout, which in turns makes direct comparison very difficult; however, it appears that the prevalence of burnout in this study was similar to Almalki *et al.* (2017) in KSA, probably higher than Al-Alawi *et al.* (2019) in KSA, Albalawi *et al.* (2015) in KSA, Stein and Sibanda (2016), Popa-Velea *et al.* (2017) and Dewitt *et al.* (2016). Our estimate also looks to be lower than Chin *et al.* (2016), Atlam (2018) and Aboalshamat (2017) (Table 7).

Our results indicated that the prevalence of burnout was higher for females 64.1% than for males 56.2%, and the three burnout dimensions are higher for females than for males. Many studies have documented gender as a risk factor for burnout (Altannir *et al.*, 2019; Chunming *et al.*, 2017). Altannir *et al.* (2019) argued that higher levels of burnout among female in KSA resulted from cultural, social and religious factors that affect the three dimensions of burnout. Traditionally females were more susceptible to stress, depression and hence to burnout.

In the present study, there was a significant association between the age of the students and client-related burnout, while the same variable is negatively associated with personal burnout dimension. In some studies, age was found to be significantly associated with burnout. Dyrbye *et al.* (2006), showed that senior medical years are associated with more significant burnout. Another study in Pakistan found that age was significantly associated with burnout (Muzafar *et al.*, 2015). The possible explanation for that is that increase in age is associated with higher academic years.

Looking for the association between the burnout syndrome and academic year, literature revealed a controversial outcomes, some studies reported a higher prevalence of burnout among students in advances clinical years (Muzafar, *et al.*, 2015; Fares *et al.*, 2016a, 2016b; Cecil *et al.*, 2014; Seo *et al.*, 2015), whereas others observed the reverse relationship (Dyrbye *et al.*, 2009). In the present study, we reported a significant correlation between the year of study and personal and work-related burnout syndrome.

In our study, only 12.9% of students reported that they always sleep for 8–9 h. Moreover, the multivariate analysis suggested a negative association between sleep duration (8–9 h) and personal and client-related burnout, although no significant association was observed. Sleep problems have been regarded as the most common symptoms of burnout. We assessed the association between sleep and burnout using one question and it may be better be assessed using any objective sleep disorder scale.

To minimize the adverse effects of burnout among the future physicians, protective strategies have been proposed in the literature, such as adequate sleep, physical activity, psychological support, educational strategies, and a better learning environment (Fares *et al.*, 2016a, 2016b). The intervention strategies may also extend to reducing the weight of daily college activities, introducing extracurricular programs and educating students about means of minimizing personal stress. Educators and decision-makers can create methods to increase the confidence and personal motivation of students with the central purpose of increasing empathy and enjoyment in the study, thus fostering healthy well-being as a substantial individual factor of protection (Dyrbye *et al.*, 2009; Fares *et al.*, 2016a, 2016b; Prins *et al.*, 2008; Youssef, 2016; Dyrbye and Shanafelt, 2016).

Although this study is the first study to investigate the burnout syndrome among medical students at our University, our research suffers from some limitations. First, our research design is a cross-sectional study which means the difficulty to determine the cause and effect and hence the relationship between the dependent variable burnout score and the set of explanatory variables should be understood in this context. Second, the comparisons of our outcomes with other studies may be affected by the mere heterogeneous nature of the studies regarding burnout as different scales were used which in turns challenges our ability to assess our results.

## Conclusions

In conclusion, our study showed that burnout syndrome was highly prevalent among medical students at Jazan University. The present study identified several factors associated with burnout in Jazan medical students. It is necessary to implement strategies to reduce the incidence of burnout among medical students for the sake of a better quality of life for future doctor.

## Figures and Tables

**Table 1:** Socio-demographic characteristics of the study participants

**Characteristics**	Male N (%)	Female N (%)	Total N (%)
***Age groups (n= 428)***			
**19-21**	89 (44.1)	94 (41.6)	183 (42.8)
**22-23**	76 (37.6)	92 (40.7)	168 (39.3)
**24-27**	37 (18.3)	40 (17.7)	77 (18.0)
***Marital status (n= 436)***			
**Single**	198 (97.1)	203 (87.5)	401 (92.0)
**Married**	6 (2.9)	26 (11.2)	32 (7.3)
**Divorced & widow**	0 (0.0)	3 (1.3)	3 (0.7)
***College level (n = 436)***			
**2nd year**	75 (36.8)	78 (33.6)	153 (35.1)
**3rd year**	48 (23.5)	43 (18.5)	91 (20.9)
**4th year**	33 (16.2)	41 (17.7)	74 (17.0)
**5th year**	27 (13.2)	42 (18.1)	69 (15.8)
**6th year**	21 (10.3)	28 (12.1)	49 (11.2)
***Grade Points Average (n= 346)***			
**Pass**	8 (4.8)	14 (7.7)	22 (6.4)
**Good**	61 (37.0)	76 (42.0)	137 (39.6)
**Very Good**	40 (24.2)	49 (27.1)	89 (25.7)
**Excellent**	56 (33.9)	42 (23.2)	98 (28.3)
***Place of Residence (n = 425)***			
**Urban**	89 (44.1)	110 (49.3)	199 (46.8)
**Rural**	113 (55.9)	113 (50.7)	226 (53.2)
**Total**	205 (100)	232 (100)	438 (100)

**Table 2:** Knowledge score about burnout according to some selected variables

	Knowledge scores			
**Variable**	**Low**	Moderate	High	*p*-value
***Age (years)***				
**19--21**	38 (21.6)	74 (42.0)	64 (36.4)	0.166
**22-23**	46 (28.0)	68 (41.5)	50 (30.5)	
**24-27**	25 (36.8)	23 (33.8)	20 (29.4)	
***Gender***				0.000
**Male**	71 (35.7)	75 (37.7)	53 (26.6)	
**Female**	40 (18.4)	94 (43.3)	83 (38.3)	
***Levels***				0.368
**2nd**	28 (18.9)	64 (43.2)	56 (37.8)	
**3rd**	27 (30.7)	35 (39.8)	26 (29.5)	
**4th**	21 (29.2)	30 (41.7)	21 (29.2)	
**5th**	21 (31.8)	27 (40.9)	18 (27.3)	
**6th**	13 (31.7)	13 (31.7)	15 (36.6)	
***GPA***				
**Pass**	8 (36.4)	10 (45.5)	4 (18.2)	0.573
**Good**	37 (28.2)	51 (38.9)	43 (32.8)	
**Very Good**	20 (23.5)	33 (38.8)	32 (37.6)	
**Excellent**	25 (26.6)	43 (45.7)	26 (27.7)	
***Place of residence***				0.512
**Urban**	49 (25.9)	72 (38.1)	68 (36.0)	
**Rural**	61 (28.2)	89 (41.2)	66 (30.6)	
**Overall Knowledge**	111 (26.7)	169 (40.6)	136 (32.7)	

**Table 3:** Prevalence of burnout among medical students based on CBI according to some selected factors

**Characteristics**	Burnout levels		*p* value
	**Free/minimal burnout**	Significant burnout	
***Gender***			0.093
**Male**	89 (43.8)	114 (56.2)	
**Female**	80 (35.9)	143 (64.1)	
***Age groups***			0.186
**19–21**	67 (38.1)	109 (61.9)	
**22–23**	61 (36.7)	105 (63.3)	
**24–27**	37 (48.7)	39 (51.3)	
***Marital status***			0.624
**Single**	158 (40.4)	233 (59.6)	
**Married**	10 (32.3)	21 (67.7)	
**Divorced & widow**	1 (33.3)	2 (66.7)	
***College level***			0.574
**2nd year**	57 (38.3)	92 (61.7)	
**3rd year**	38 (43.2)	50 (56.8)	
**4th year**	30 (40.5)	44 (59.5)	
**5th year**	21 (31.8)	45 (68.2)	
**6th year**	22 (45.8)	26 (54.2)	
***Grade Points Average***			0.360
**Pass**	9 (40.9)	13(59.1)	
**Good**	46 (34.1)	89 (65.9)	
**Very Good**	38(43.2)	50 (56.8)	
**Excellent**	42 (44.7)	52 (55.3)	
***Place of Residence***			0.216
**Rural**	93 (41.9)	129 (58.1)	
**Urban**	69 (35.9)	123 (64.1)	
**Overall Prevalence** **95% C.I**	169 (39.8)	256 (60.2) 55.6 –64.8	

**Table 4:** CBI burnout scores, mean (SD) according to burnout dimensions among the medical students, according to some selected factors

**Characteristics**	CBI burnout scores, mean (SD)		
	**Personal burnout**	Study-related burnout	Client-related burnout-
***Gender***			
**Male**	67.7 (18.2)	59.3 (17.2)	44.7 (22.9)
**Female**	70.9 (22.0)	61.9 (19.6)	37.6 (23.7)
***p* value**	0.115	0.155	0.002
***Age groups***			
**19–21**	70.6 (20.6)	58.8 (19.8)	37.9 (24.1)
**22–23**	69.5 (19.8)	62.2 (17.4)	43.4 (23.1)
**24–27**	66.6 (20.8)	60.8 (18.0)	43.0 (22.6)
***p* value**	0.358	0.250	0.076
***Marital status***			
**Single**	72.8 (21.4)	64.8 (20.6)	37.6 (19.3)
**Married**	70.8 (31.5)	76.8 (2.5)	23.3 (22.5)
**Divorced & widow**	69.0 (20.2)	60.2 (18.4)	41.5 (23.9)
***p* value**	0.601	0.200	0.289
***College level***			
**2nd year**	70.6 (20.9)	58.7 (18.6)	35.8 (23.5)
**3rd year**	68.9 (19.6)	61.2 (19.0)	44.2 (20.8)
**4th year**	67.0 (20.2)	60.9 (18.7)	44.2 (25.7)
**5th year**	72.3 (19.5)	64.7 (18.5)	42.2 (23.0)
**6th year**	67.0 (20.9)	60.3 (17.2)	44.2 (24.5)
***p* value**	0.464	0.307	0.026
***Grade Points Average***			
**Pass**	67.9 (22.1)	60.2 (17.5)	41.0 (21.0)
**Good**	71.2 (21.7)	63.9 (19.2)	42.8 (24.1)
**Very Good**	67.0 (18.7)	57.4 (18.1)	38.0 (22.1)
**Excellent**	68.2 (19.7)	57.3 (18.1)	43.0 (25.4)
***p* value**	0.470	0.026	0.453
***Place of Residence***			
**Urban**	72.2 (19.5)	62.8 (18.9)	42.3 (24.7)
**Rural**	67.8 (20.5)	60.0 (17.3)	40.2 (22.5)
***p* value**	0.028	0.126	0.367
**All Students**	69.5 (20.3)	60.7 (18.6)	41.0 (23.6)

**Table 5:** Preventive measures among the study participant regarding the burnout

**Statement**	Always	Often	Sometimes	Rarely	Never	Mean (D)
**Having a full day to do what you like**	45 (10.4)	94 (21.7)	181 (41.7)	98 (22.6)	16 (3.7)	3.12 ± 0.99
**Having time for your Self**	120 (27.6)	158 (36.4)	112 (25.8)	39 (9.0)	5 (1.2)	3.80 ± 0.98
**Having a good vacation.**	108 (24.9)	76 (17.6)	74 (17.1)	73 (16.9)	102 (23.6)	3.03 ± 1.51
**Practicing exercise for at least one hour**	26 (6.0)	65 (15.0)	98 (22.7)	139 (32.2)	104 (24.1)	2.46 ± 1.18
**Doing Something for fun**	120 (27.7)	144 (33.3)	131 (30.3)	30 (6.9)	8 (1.8)	3.78 ± 0.98
**Having time for friends and family**	71 (16.5)	132 (30.6)	160 (37.1)	57 (13.2)	11 (2.6)	3.45 ± 0.99
**Share your stress with others**	74 (17.1)	109 (25.1)	119 (27.4)	71 (16.4)	61 (14.1)	3.14 ± 1.28
**Good sleep quality ( 8-9 hours per night)**	56 (12.9)	97 (22.4)	143 (32.9)	88 (20.3)	50 (11.5)	3.04 ± 1.18
**Say “No!” to inappropriate things**	91 (21.0)	128 (29.6)	149 (34.4)	52 (12.0)	13 (3.0)	3.53 ± 1.04
**Feeling a healthy Life**	28 (6.5)	69 (15.9)	148 (34.2)	123 (28.4)	65 (15.0)	2.70 ± 1.10

**Table 6:** Regression analysis of the factors associated with burnout

**Predictors**	Burnout dimensions					
	**Personal burnout**		Study-related burnout		Client--related burnout	
	**Beta**	*p* value	Beta	*p* value	Beta	*p* value
**Having a good vacation.**	−0.610	0.148	−0.010	0.798	0.093	0.169
**Practicing exercise for at least one hour**	0.024	0.944	0.022	0.624	0.006	0.613
**Doing Something for fun**	0.092	0.696	0.055	0.478	−0.086	0.391
**Having time for friends and family**	0.907	0.069	−0.027	0.704	0.023	0.030
**Share you're stress with others**	−0.850	0.064	0.046	0.359	0.028	0.054
**Good sleep quality (8-9 hours per night)**	−0.429	0.159	0.027	0.619	−0.176	0.514
**Say “No!” to inappropriate things**	0.598	0.075	−0.001	0.989	−0.104	0.870
**Feeling a healthy Life**	0.709	0.074	−0.071	0.170	0.037	0.070
**Knowledge Score**	0.710	0.025	0.277	0.000	−0.017	0.660
**Age**	−30.17	0.026	–	–	0.762	0.002
**Gender (female)**	−0.896	0.016	0.136	0.016	−0.103	0.265
**Academic year**	2.795	0.025	0.175	0.000	0.115	0.130
**CGPA**	−1.030	0.047	0.369	0.000	0.305	0.037
***R^2^***	0.96		0.90		0.77	

**Table 7:** Prevalence of burnout among students during the past five years

**Study**	Sample size	Population	Country	Scale	Prevalence(%)
**Al-Alawi *et al.* (2019)**	662	Sultan Qaboos University	Oman	MBI*	7.4
**Albalawi *et al.* (2015)**	140	Tabuk University	KSA	MBI	48.6
**Almalki *et al.* (2017)**	249	King Saud bin Abdulaziz University for Health Sciences	KSA	MBI	61.8
**Stein and Sibanda (2016)**	93	University in Johannesburg	South Africa	CBI	31.
**Chin *et al.* (2016)**	452	Universiti Sains Malaysia	Malaysia	CBI	67.9.
**Atlam (2018)**	672	Tanta University	Egypt	CBI	79.9
**Fares *et al.* (2016a)**	165	Private university in Beirut,	Lebanon	MBI	75.2
**Popa-Velea *et al.* (2017)**	299	the University of Medicine in Bucharest	Romania	MBI	15.05
Dewitt *et al.* (2016)	688	five Australian medical schools	Australia	CBI	51
**Aboalshamat *et al.* (2017)**	645	medical and dental students in Jeddah	KSA	CBI	67.9

Note: MBI: Maslach Burnout Inventory-Educators Survey (Maslach *et al.*, 1996)
